# Biodiversity in agricultural landscapes: The effect of apple cultivar on epiphyte diversity

**DOI:** 10.1002/ece3.2683

**Published:** 2017-01-26

**Authors:** C. Robin Stevenson, Chantel Davies, Jennifer K. Rowntree

**Affiliations:** ^1^ King's LynnNorfolkUK; ^2^ Growing Research InternationalCoventryUK; ^3^ Centre for the Genetics of Ecosystem ServicesFaculty of Life SciencesUniversity of ManchesterManchesterUK

**Keywords:** apple orchards, bryophyte, community genetics, extended phenotype, intraspecific genetic variation, productive landscapes

## Abstract

In natural systems, extended phenotypes of trees can be important in determining the species composition and diversity of associated communities. Orchards are productive systems where trees dominate, and can be highly biodiverse, but few studies have considered the importance of tree genetic background in promoting associated biodiversity. We tested the effect of apple cultivar (plant genetic background) on the diversity and composition of the associated epiphytic bryophyte community across a total of seven cultivars in five productive East Anglian orchards where each orchard contained two cultivars. Data were collected from 617 individual trees, over 5 years. Species richness and community composition were significantly influenced by both orchard and cultivar. Differences among orchards explained 16% of the variation in bryophyte community data, while cultivar explained 4%. For 13 of the 41 bryophyte species recorded, apple cultivar was an important factor in explaining their distribution. While the effects of cultivar were small, we were able to detect them at multiple levels of analysis. We provide evidence that extended phenotypes act in productive as well as natural systems. With issues of food security ranking high on the international agenda, it is important to understand the impact of production regimes on associated biodiversity. Our results can inform mitigation of this potential conflict.

## 1 Introduction

A recurring theme in ecology is that patterns of species' distributions and abundances are shaped not only by environmental factors, but also by interactions with other organisms (Thompson, 2013). It is now well documented that genetic diversity and genetic identity within a focal species can play an important role in determining the composition and diversity of associated communities (Crutsinger, 2016; Hughes, Inouye, Johnson, Underwood, & Vellend, 2008; Rowntree, Shuker, & Preziosi, 2011). Much of the evidence for these “community genetic” or “extended phenotype” effects comes from forests, where genotypic variation within tree species has been associated with changes in, among other things, arthropod (Bangert et al., 2006; Barbour, Forster, Baker, Steane, & Potts, 2009a), soil microbial (Schweitzer et al., 2008) and epiphyte community diversity (Zytynska, Fay, Penney, & Preziosi, 2011) and abundance (Lamit et al., 2011). While the importance of within‐species genetic variation in structuring ecological communities has been demonstrated both experimentally (Johnson & Agrawal, 2005) and in the wild (Zytynska et al., 2011), questions remain as to the relative importance of these “community genetic effects” compared to the other causal factors in the local environment (Hersch‐Green, Turley, & Johnson, 2011). Of particular relevance is work that has shown the “dilution” of host‐plant genetic effects on associated communities at increasing spatial scales (Tack, Johnson, & Roslin, 2012; Tack, Ovaskainen, Pulkkinen, & Roslin, 2010). In addition, although genetic diversity and genetic identity of focal species is often tightly controlled in agricultural landscapes, there has been limited focus on the ecological relevance of community genetic effects in such intensively managed habitats. Productive forest plantations make ideal seminatural laboratories in which to address these questions, as multiple cultivated varieties (cultivars) or natural genetic varieties are often planted together and at multiple geographic locations across a landscape (Barbour et al., 2009b; Dutkowski & Potts, 1999). Forestry plots of *Eucalyptus globulus* have been used to good effect in previous studies (Barbour et al., 2009b; O'Reilly‐Wapstra et al., 2014), and the apple orchards of East Anglia potentially provide such an experimental system in the UK.

A variety of different cultivars are often planted in orchards, mainly in order to cater for different sectors of the market, or as an insurance against cropping failure of any single cultivar. In addition, while some apples produce abundant fertile pollen of their own, others do not (Dennis, 2003; Jackson, 2003) and in the latter case, pollinator cultivars are planted alongside, or between, the commercial crop cultivars (Jackson, 2003). This means that many apple orchards contain multiple cultivars of the same age growing together (Roach, 1956) under identical environmental conditions.

Epiphytic bryophytes will naturally colonize the trunks and branches of apple trees, and, historically, applications of “tar oil” (coal tar distillate) were used to kill any epiphytes that grew, as they were thought to harbor pests (Morgan & Marsh, 1956; Weathers, 1913). This practice ceased, however, in the mid‐1970s. Thus, most of the current epiphyte flora of apple trees in the UK has become established over the past 40 years (personal communication from local growers). Epiphytic plants grow on, but do not parasitize, other plants (Benzing, 1990). Bryophytes, *that is,* mosses and liverworts, are common epiphytes on trees and are often the only epiphytic plants in temperate regions (Bates, 2009; Smith, 1982). Bryophytes are important primary producers in forest systems, contributing to carbon fixation and nitrogen cycling (Longton, 1992; Turetsky, 2003), and can act as indicators of environmental quality (Hejcman et al., 2010). Like epiphytes in general, their distribution is determined by a number of abiotic and biotic factors. These include characteristics of the host, such as bark roughness, size (González‐Mancebo, Losada‐Lima, & McAlister, 2003), and pH (Lewis & Ellis, 2010; Whitelaw, 2012), as well as forest structure (Király & Ódor, 2010) and microclimate (Mota de Oliveira, ter Steege, Cornelissen, & Robbert Gradstein, 2009; Sporn, Bos, Kessler, & Gradstein, 2010).

The value of orchards for biodiversity in the UK has been increasingly recognized since the designation of traditional orchards as priority habitats for the UK Biodiversity Action Plan initiative (Wedge & Robertson, 2010). This initiative has emphasized the value of lesser‐studied groups, such as bryophytes and lichens (Lush et al., 2009; Robertson, Marshall, Slingsby, & Newman, 2012). Previous work on orchard biodiversity has often focused on differences in management practice, and, in particular, the distinction between traditional and intensive management (Robertson et al., 2012). Our aims with this work were to assess the epiphytic diversity of productive East Anglian orchards under conventional management and to investigate whether apple cultivar was also a factor in determining epiphyte community composition. We sampled at multiple locations enabling us to investigate the relative importance of cultivar in supporting a diverse epiphyte community, in the context of different environmental and management conditions.

## 2 Materials and Methods

**Figure 1:**
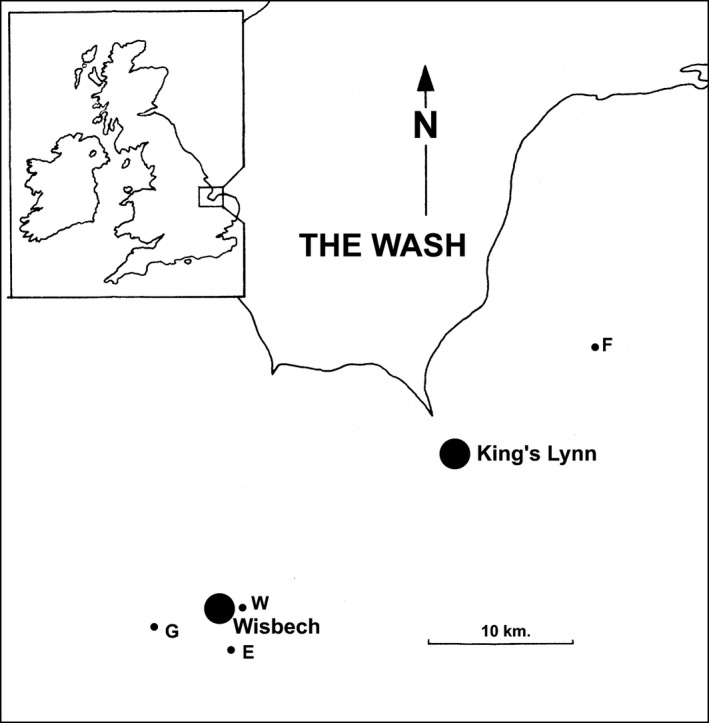
The study area in relation to its position within the UK. Locations of the five orchards (Elm [E], Flitcham A & B [F], Gorefield [G], Walsoken [W]) are shown in relation to the East Anglian towns Wisbech and King's Lynn

We surveyed five apple orchards (Table 1; Figure 1) in East Anglia between 2005 and 2009 for epiphytic bryophytes. Two of the orchards were owned and managed by a single company and were in close proximity to each other (Flitcham A and Flitcham B). They were however distinct plantings, containing different combinations of cultivars, and were therefore included as separate units in the analyses. The trees in each orchard were mainly maintained as half standards, that is, pollarded at a height of about a meter, and under similar management regimes. This means that an examination of the whole tree was possible as all trees allowed easy access to the canopy, as well as the trunk and lower branches. The Bramleys and Howgate Wonders are longer‐lived trees and so have thicker trunks and branches but the bryoflora of the entire tree remain accessible. Management included the application of ground and foliar nitrogen fertilizers, ground herbicides, regular spraying with fungicides, pheromone trapping of invertebrates, and control with suitable pesticides (personal communication from local growers). This differed somewhat among orchards accounting for some of the among orchard variation in the data.

**Table 1:** Locations of orchards, survey date, and cultivar information

Orchard (County)	OS grid/Lat‐Long	Year surveyed	Cultivars (number surveyed)	Distribution of cultivars	Year of planting (age when surveyed)
Walsoken (Norfolk)	TF475093 52°40′23′′N 0°11′′58′′E	2005	Bramley (50) Howgate Wonder (50)	Planted in alternate rows	1968 (37)
Gorefield (Cambs.)	TF405091 52°39′45′′N 0°04′41′′E	2006	Bramley (50) Grenadier (50)	Grenadiers planted as pollinators approx. every 3rd tree per row	1968 (38)
Elm (Cambs.)	TF460066 52°38′15′′N 0°09′24′′E	2007	Bramley (50) Lord Derby (50)	Lord Derbys planted as pollinators; approx. every 3rd tree per row	1965 (42)
Flitcham A (Norfolk)	TF721280 52°49′19′′N 0°33′11′′E	2006	Cox (100) Fortunes (100)	Planted as large separate, but adjacent, blocks	1956 (50)
Flitcham B (Norfolk)	TF720280 52°49′21′′N 0°33′038′′E	2009	Cox (58) Worcester (59)	Worcesters planted as pollinators: every 3rd tree in every 3rd row	1956 (53)

Within each orchard, we surveyed two cultivars. Chosen cultivars were planted at the same time either in adjacent blocks, or interspersed in a single block, when one was a pollinator (see Table 1 for more information). At least 50 trees per cultivar (maximum 100) were examined in detail at a rate of approximately 25 trees per day. Each tree was subjected to a 360° examination, branch by branch, and a list of all the epiphytic bryophytes occurring made, although no attempt was made to record bryomass. Bryophytes were separated into mosses and liverworts and defined as obligate or facultative epiphytes. Obligate epiphytes were those species, which occur most frequently as epiphytes throughout the region studied. This differs slightly from the definitions provided by Bates, Proctor, Preston, Hodgetts, and Perry (1997). Facultative epiphytes were those species that are also commonly found on other substrata (*e.g.,* soil or rocks) in the area. No individual tree was surveyed more than once. Bryophyte nomenclature follows Hill, Blackstock, Long, and Rothero (2008), and species were identified by C. Robin Stevenson (CRS).

### 2.1 Data analysis

All analyses were undertaken in the R statistical programming environment, version 3.2.3 (R Core Team 2015) and graphics produced using the “ggplot2” package (Wickham, 2009).

### 2.2 Descriptive statistics

Species richness was calculated as the total number of different epiphyte species per individual tree. These data were analyzed using a general linear model where orchard was included as a main effect and cultivar was nested within orchard. Residuals from this model were normally distributed, and hence, it was chosen over a model with a Poisson distribution. Significance values were calculated using type II tests in the ANOVA function in the “car” package (Fox & Weisberg, 2011).

### 2.3 Epiphytic bryophyte community composition

The species composition of the epiphytic bryophyte communities was explored using multivariate statistics. Data were first cleaned by removing duplicate lines in the species matrix (*i.e.,* where the bryophytes observed on different trees were exactly the same) and trees where no bryophytes were recorded. This reduced the data set to a total of 538 trees with most of the removals coming from the Howgate Wonders, leaving eight trees for this cultivar. Data were then transformed using the double Wisconsin transformation as recommended by Oksanen (2015) and a Jaccard distance matrix constructed. The bryophyte community composition of each tree was explored visually using a nonmetric multidimensional scaling ordination (NMDS) in the “metaMDS” package where the results presented are the best of 20 random analyses. Permutation tests (10,000 randomizations) were performed in the “adonis” package where the effect of orchard was first estimated on the distance matrix followed by the effect of cultivar. An additional analysis was run where location (near Wisbech or Flitcham) was also included in the model. All community analyses were undertaken using the “vegan” package (Oksanen et al., 2015).

### 2.4 Species‐level effects

Due to the highly unbalanced data and high abundance of zeros, we used a random effects only generalized linear mixed model with a binomial distribution and a logit link function in the package “lme4” (Bates, Maechler, Bolker, & Walker, 2014) to test the effect of orchard and apple cultivar on the presence and absence of each bryophyte species. A null model, which specified orchard as a random factor, was tested for significance against a full model that included apple cultivar nested within orchard as the random factor. Where cultivar nested within orchard was a significantly better fit, relative variance was calculated as the percentage variation attributed to cultivar nested within orchard compared to total variance explained by the random factors. Significance values were obtained by likelihood ratio tests of the null model against the full model. These were adjusted for multiple pairwise comparisons using the function “p.adjust” in package “stats” using the Benjamini and Hochberg (1995) false discovery rate (FDR). Data were not split by the two main locations for these analyses as this reduced the power and nesting of cultivar within orchard should account for differences among cultivars within each orchard.

## 3 Results

A total of 41 bryophyte species (38 mosses, three liverworts) were found to be growing epiphytically on the apple trees across all five orchards surveyed (Appendix S1). Of these, 19 were obligate epiphytes and 22 facultative epiphytes. The cultivar Howgate Wonder was characterized by a distinct lack of epiphytes compared to the other cultivars.

**Figure 2:**
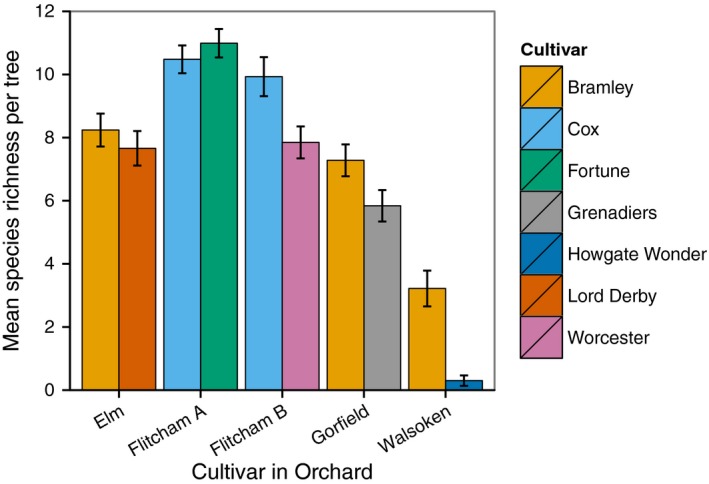
Mean species richness per tree in the five orchards surveyed. Orchard is shown on the *x*‐axis, and apple cultivars are different colors. Error bars are 95% confidence intervals

There was a highly significant effect of orchard on epiphyte species richness per tree (*F*_4,607_ = 358.17, *p* < 2.2 × 10^−16^) and a highly significant effect of cultivar nested within orchard (*F*_5,607_ = 20.92, *p* < 2.2 × 10^−16^; Appendix S2). Mean species richness of epiphytes per tree was highest at Flitcham A (Cox: Mean = 10.48, *SE* = 0.22; Fortune: Mean = 10.99, *SE* = 0.23) and lowest at Walsoken (Bramley: Mean = 3.22, *SE* = 0.28; Howgate Wonder: Mean = 0.30, *SE* = 0.08). The cultivar Bramley occurred in three of the orchards surveyed, and the number of epiphytes per tree supported by this cultivar was highest at Elm (Mean = 8.24, *SE* = 0.26) and the lowest at Walsoken (Figure 2).

### 3.1 Community Composition

**Figure 3:**
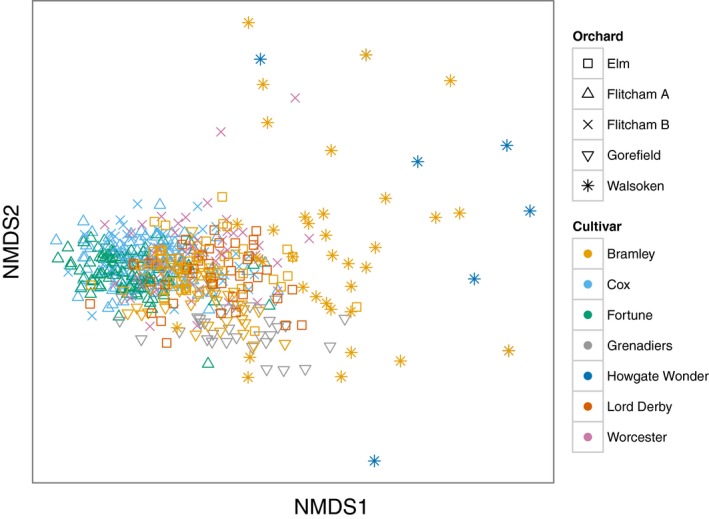
Nonmetric multidimensional scaling ordination plot showing the similarity of epiphytic bryophyte communities on individual apples trees. Individual points show trees, orchards are denoted by different symbols, and apple cultivars, by different colors. Stress = 0.2. Permutation tests showed orchard to explain 16% of the variation in the data and cultivar 4%

The NMDS did not converge after 20 attempts, so the best solution is presented with stress values of 0.2 (Figure 3, Appendix S4). Permutation tests showed that orchard explained 16% of the variation in epiphytic bryophyte community composition (*F*_4,528_ = 26.36, *p* = 0.0001) and cultivar (*F*_5,528_ = 5.55, *p* = 0.001) explained 4% of the variation. Therefore, orchard explained four times as much variation in the data as cultivar, and 80% of the variation in the data remained unexplained by the model (Appendix S3). When location was included in the model, the amount of variation explained by cultivar remained the same, but the variation explained by orchard in the first model was split evenly between location (8%) and orchard (8%).

### 3.2 Species‐level effects

Cultivar nested within orchard was important in explaining the presence or absence of 13 (32%) species, and, of these, 11 were facultative and two were obligate epiphytes (Table 2). The presence or absence of the remaining 28 species was best explained by orchard alone. For three species (*Grimmia pulvinata*,* Kindbergia praelonga, and Metzgeria furcata*), cultivar nested within orchard accounted for more than 50% of the variation explained by the model. Of these, *G. pulvinata* and *K. praelonga* are facultative epiphytes and were present on at least one tree in all orchards and on all cultivars. *Metzgeria furcata* is an obligate epiphyte and was only observed on the Fortune cultivar at Flitcham A and on the Grenadier cultivar at Gorefield. For *Ceratodon purpureus*, around 50% of the variation explained was attributed to cultivar nested within orchard. This is another facultative species that was present in all orchards and on all cultivars except for Howgate Wonder at Walsoken (Appendix S1).

**Table 2:** Data from the GLMM model for the individual species where cultivar was an important factor in explaining their distribution, with corresponding Chi‐square, adjusted *p* values and the relative variance explained by cultivar nested within orchard

Species	Epiphyte	χ^2^ value	*p* value	Relative variance (orchard/cultivar) (%)
*Amblystegium serpens*	Facultative	15.57	2.8 × 10^−03^	15
*Brachythecium rutabulum*	Facultative	28.96	3.0 × 10^−06^	33
*Bryum capillare*	Facultative	12.73	1.2 × 10^−02^	18
*Ceratodon purpureus*	Facultative	11.74	1.9 × 10^−02^	47
*Dicranoweisia cirrata*	Facultative	19.39	3.8 × 10^−04^	36
*Grimmia pulvinata*	Facultative	28.82	3.1 × 10^−06^	70
*Hypnum cupressiforme agg*.	Facultative	11.65	1.9 × 10^−02^	21
*Kindbergia praelonga*	Facultative	72.41	9.2 × 10^−15^	87
*Metzgeria furcata*	Obligate	11.77	1.9 × 10^−02^	100
*Orthotrichum affine*	Obligate	27.89	4.8 × 10^−06^	30
*Orthotrichum diaphanum*	Facultative	50.21	5.7 × 10^−11^	36
*Rhynchostegium confertum*	Facultative	12.75	1.2 × 10^−02^	31
*Zygodon viridissimus*	Facultative	28.49	3.6 × 10^−06^	5

## 4 Discussion

We investigated the influence of orchard and cultivar at three different levels of epiphyte diversity in conventionally managed UK apple orchards. First, we used summary species richness statistics to look at the overarching effects of orchard and cultivar. Next, we explored their impact on the epiphyte community composition and finally determined the importance of these factors for individual epiphyte species. In all cases, both orchard and cultivar were important in explaining the levels of epiphyte diversity found. The factor orchard encompassed a complex variety of factors including location, microclimate, and management. As expected, and at all levels of analysis, this factor explained more variation in the data than cultivar. However, the consistency of the cultivar effect leads us to conclude that genetic background of the tree is a small but important factor in determining the diversity and composition of associated epiphyte communities in apple orchards.

### 4.1 Artificial levels of genetic diversity

Previous studies have highlighted the fact that in many experiments where the importance of within‐species genetic diversity has been explored, the levels of genetic diversity tested are artificially high (Tack et al., 2012). This comes about as divergent genotypes from across a landscape have been clustered together to form artificial interacting communities, where intraspecific differences are emphasized. This is not universally the case, however, and other studies have found natural levels of within‐species genetic diversity to be an important factor in structuring associated ecological communities (Davies, Ellis, Iason, & Ennos, 2014; Zytynska et al., 2011). The apple orchards of East Anglia are, due to their horticultural origins, “artificial” woodland; however, they are relatively stable features of the landscape. The life span of a commercial orchard can be up to 60+ years if properly managed, and the ages of the orchards we sampled ranged from 37 to 53 years. While there has been much interest in the value of orchards for their biodiversity in recent years, much of this focus has been on traditionally managed orchards (Lush et al., 2009; Wedge & Robertson, 2010). Here, we provide evidence that conventionally managed orchards also have a biodiversity value and that the cultivars planted within these will have an impact on the associated biodiversity that they can support.

### 4.2 Orchard effects

At a regional level, all the orchards studied experience the same climatic conditions, as they are situated within a maximum of 40 km of each other. That said, microclimatic conditions will inevitably vary among sites. All sites visited are protected, to a greater or lesser extent, by hedges. These range from tall dense conifer belts around the perimeter, through to external (and sometimes internal) plum hedges. The only site not so protected was Walsoken, where the hedges are replaced, on at least one side, by housing.

The most obvious differences among the sites relate to their aspect, elevation, and soils, in particular between the Flitcham orchards and those around Wisbech. Inclusion of these location clusters in the community analyses suggests that around half of the variation among orchards is indeed attributable to this distinction. The Flitcham orchards are located on gentle west facing slopes lying between 70 and 50 m above sea level, while the orchards centered around Wisbech (Figure 1) lie at about 3 m above sea level, roughly translating to a temperature difference of −0.2°C. In addition, it is possible that cold air drainage could give the sites around Wisbech lower overnight minima than the sloping Flitcham, although without accurate records this is would be difficult to quantify. The soils at Flitcham consist of “typical brown calcareous loams and sands” (Soil Survey of England and Wales 1979)**,** while those round Wisbech consist of brown warp soils (very fine sandy loams, silty loams, silty clay loams, and silty clays of estuarine origin (Perrin & Hodge, 1965)). Soil nutrients are known to influence overall tree chemistry (Gustafsson & Eriksson, 1995), and this could affect epiphyte distribution (Whitelaw, 2012), although it remains unclear how important soil nutrients specifically might be in relation to the distribution of epiphytic bryophytes.

If half of the variation attributed to orchard can be explained by the differences among the location clusters, the remaining variation is likely due to differences in management practice. Planting patterns differed among orchards (Table 1) and could influence the local dispersal and colonization abilities of the bryophytes onto different cultivars. In apple orchards, the planting pattern is determined by the characteristics of the cultivars and their ability to self‐pollinate. Where the crop produced by the pollinator species is of little or no commercial value, the planting pattern minimizes their presence and pollinators will be planted as single trees surrounded by the main cultivar of interest (Roach, 1956). This isolates them, making dispersion of bryophytes among cultivars of this type more problematic, and should act to minimize differences in the epiphyte community with the surrounding (crop) trees. Our data do not support this supposition as we found clear differences between the cultivars in two of the three orchards where the pollinators were surrounded by the crop (Gorefield and Flitcham B). In addition at Flitcham A, where the planting pattern of separate adjacent blocks for the two cultivars should work to maximize differences among cultivars, little difference was found between the epiphyte communities of the cultivars surveyed. These data suggest that planting patterns did not greatly influence the distribution of epiphytes.

Other management practices, such as pruning and chemical application, likely differed among the sites sampled as well. Various chemicals are applied to the trees in apple orchards in order to control pests and diseases (Beers, Suckling, Prokopy, & Avila, 2003; Grove, Eastwell, Jones, & Sutton, 2003; Jackson, 2003). The most important, in terms of the epiphyte community, is probably the application of fungicides. This has been shown to have an adverse effect on epiphytic lichens, which, in turn, may actually benefit the bryophytes by reducing competition (Bartok, 1999). Tar oil used to be frequently applied to orchard trees specifically to remove epiphytic flora (Morgan & Marsh, 1956; Weathers, 1913). In the orchards we visited, this practice ceased in the mid‐1970s (personal communication local growers) and it seems unlikely that there would be any direct legacy effect of this application on the epiphyte flora surveyed. Tree age differed among orchards ranging between 37 and 53 years at the time of surveying, and previous studies have shown that age can influence epiphyte community (Snäll, Ehrlén, & Rydin, 2005). The cessation of tar oil applications at a similar time in our orchards serves to reduce any differences caused by the age range of the trees as, in effect, the epiphyte community could only become established once this practice had stopped. Therefore, the effective age of the trees surveyed in this study, in terms of the length of epiphyte colonization time, ranged from around 30 to 36 years. Some of the effects of tree age, however, are thought to be due to changes in bark texture, with increasing fissuring with age encouraging epiphyte colonization (Gustafsson & Eriksson, 1995; Lamit et al., 2015). These differences would obviously still remain; however, most of the epiphytes we recorded were found on the branches of the trees where fissuring is less apparent (CRS, personal observation).

Pruning practices differ chiefly due to factors such as the availability of skilled labor and financial constraints. Differences in pruning intervals and intensity will undoubtedly have an effect on epiphytic bryophyte populations, as frequent pruning (to increase fruit yield) will open up the canopy, and pruning, in general, affects the shape of the tree (Roach, 1956). Removing branches and opening up the canopy changes the surface water dynamics of the trees, altering stem flow patterns, resulting in potentially less humid surfaces (Jackson, 2003), and therefore, less suitable habitats for some bryophyte species. In addition, pruning can expose new surfaces for colonization, thus potentially changing the successional dynamics of the epiphyte community.

### 4.3 Cultivar effects

The impact of cultivar on the epiphyte community can best be visualized using the species richness data from the orchards Flitcham B, Gorefield, and Walsoken. In Flitcham B, Cox supported a greater number of epiphyte species than Worcester, and at Gorefield and Walsoken, Bramley supported more epiphyte species than Grenadiers and Howgate Wonder, respectively. Howgate Wonder, in particular, was found to support fewer species, as in many cases, no epiphytes were growing on it. By comparing cultivars planted within the same orchard, we can control for some of the factors that influence differences among orchards. This is because within an orchard plantation, factors such as soil, the surrounding environment, and management practices will be more consistent across cultivars than among different orchards. Therefore, the differences seen between cultivars within a single orchard are highly likely to be due to the properties of the cultivars themselves making them more or less suitable epiphyte hosts. Differences among cultivars are the result of selective breeding by growers attempting to produce cultivars with a variety of desirable properties, such as disease resistance. More detailed analysis of the influence of specific cultivar traits on epiphyte diversity and abundance is required in order to understand these effects more fully.

Previous studies on epiphytic bryophytes have mainly focused on the role of host bark, and bark chemistry in determining their distribution (Coker, 1967; Manzke, 2008; Whitelaw, 2012). Host bark traits such as thickness (Dutkowski & Potts, 1999), the level of decortication (Barbour et al., 2009a), and roughness (Lamit et al., 2011, 2015) have been shown to be genetically determined in other tree species. These bark traits can influence the composition of associated communities of macroarthropods (Barbour et al., 2009a) and epiphytes (Lamit et al., 2011, 2015). In a study on orchard biodiversity, Whitelaw (2012) found evidence suggesting a link between tree cultivar, bark chemistry, and epiphytic bryophyte diversity. Within a single orchard, there were significant differences in the number of bryophyte species and bryophyte cover per tree when comparing two apple cultivars (Ashmead's Kernal and Newton Like). This was accompanied by significant differences in bark pH and nitrogen concentrations between the cultivars. In a related in vitro experiment, Whitelaw (2012) found that low pH inhibited spore germination and growth in a number of bryophytes species (*Brachytheciastrum velutinum*,* Rhynchostegium confertum*,* Orthotrichum affine,* and *Bryum capillare*), while high concentrations of nitrogen inhibited spore growth but not germination in the species *O. affine*. This work suggests that biochemical factors associated with tree bark traits can have an impact on the life‐history traits of epiphyte species and thus contribute to changes in community composition. We did not test bark pH or chemistry in our study, but it is likely that these factors do influence the differing distributions we see across cultivars.

Cultivars of horticultural trees are bred to possess a suite of different, genetically determined traits, some of which may also impact on the cultivar's suitability as an epiphyte host. These traits include differential susceptibility to disease, tree size, branching habit, and suitability to particular rootstocks (Jackson, 2003; Webster & Wertheim, 2003). Tree size and architectural structure are both factors known to influence epiphyte communities (McCune et al., 1997; Pentecost, 1998; Williams & Sillett, 2007), and bryophytes, in particular, are sensitive to changes in microclimate and patterns of water availability (Vanderpoorten & Goffinet, 2009), which are also influenced by differences in the architectural structure. However, management mechanisms such as pruning should serve to minimize these differences, at least within a single orchard. As tree size increases, the likelihood of finding more species also increases (Arrhenius, 1921). Two of the cultivars we sampled were larger (in terms of girth) than the rest (Bramley and Howgate Wonder); thus, we might expect elevated levels of epiphyte richness on these cultivars. While the Bramley cultivar did support relatively high levels of epiphyte species where it was found, Howgate Wonder was a poor epiphyte host, suggesting that size alone does not determine the richness of the epiphyte communities on the trees.

### 4.4 Facultative versus obligate epiphytes

We categorized the epiphytes observed as facultative and obligate species. Of the 41 species observed, 22 were facultative and 19 obligate. For the species where cultivar significantly influenced their distribution, 11 were facultative and only two obligate, suggesting that cultivar was a more important factor for the facultative than the obligate species.

Colonization and recruitment processes are obviously an important factor determining the distribution of epiphytic bryophytes. Initial colonization is likely to come from local sources, with new taxa arriving as spores, gemmae, or plant fragments (Vanderpoorten & Goffinet, 2009). Distance from a source habitat, prevailing weather, and animal vector movement patterns will all determine how successful (or swift) colonization will be (Hutsemekers, Dopagne, & Vanderpoorten, 2008), as will the suitability of the host tree. There are slight differences in the propagule sources and local availability of the facultative and obligate species, which may influence the relative importance of orchard and cultivar. Recruitment of facultative species probably comes from the spore rain, from the soil diaspore bank, or from importation by animal vectors. Recruitment of the majority of the obligate species is also from the spore rain, or via animal vectors, but unlikely to be from the soil. Therefore, the number of sources of propagules is reduced for the obligate species. In addition, the obligate species are, to a large extent, pollution‐sensitive “recolonizers.” These were adversely affected by acid rain during the 1960s causing many to disappear (Bates & Preston, 2011; Bates, Roy, & Preston, 2004; Blockeel, Bosanquet, Hill, & Preston, 2014). In contrast, the majority of the facultative species we observed are less sensitive to pollution, locally common, and freely produce sporophytes in the region. This means that there will have been a larger and more uniform pool of facultative species available to colonize apple trees once applications of tar oil ceased and air quality increased, whereas obligate species will have been more sparsely and patchily distributed. It follows that for the facultative species, local differences such as cultivar become a more important distinguishing factor in epiphyte distribution, whereas the distribution of obligate species is more likely defined by location (*i.e.,* orchard). Finally, many of the facultative species (*e.g., Hypnum cupressiforme*) possess vigorous growth forms that, once established, may limit the space available for colonization of later arriving obligate species, thus reinforcing initial patterns of colonization.

### 4.5 Conclusions

In summary, productively managed orchards can be valuable habitats for epiphytic bryophytes. Recognizing the biodiversity value of these is particularly pertinent as traditional orchards are currently declining. In our study, both the location of the orchard and cultivar planted influenced the composition of the epiphyte community present. Although orchard (location) explained more variation in the data, within‐orchard effects of cultivar remained an important factor in determining epiphytic species richness, community composition, and the presence of individual species. Cultivar was more important for the facultative bryophyte species (non‐epiphyte specialists), which is likely due to their underlying distribution in the local area and legacy effects of earlier air pollution. Therefore, the relative value of a productive orchard for biodiversity conservation will depend on the cultivars planted as well as the location and the management practices employed.

## Data accessibility

R scripts are included in Appendix S5. Data files are deposited in the Dryad Digital repository: doi:10.5061/dryad.mb0sh.

## Conflict of interest

None declared.
