# Supplementary material for: Biodiversity in agricultural landscapes: The effect of apple cultivar on epiphyte diversity
Source: Ecol Evol. 2017 Jan 26;7(4):1250–8. doi: 10.1002/ece3.2683 (PMC5306003; doi:10.1002/ece3.2683)
Supplement: Supplementary file 1 [file ECE3-7-1250-s001.docx]

**Appendices**

Appendix 1. Species list (nomenclature according to Hill *et al* 2008) and description of the epiphytic bryophytes growing on the trunks and branches of apple trees surveyed across five orchards (Flitcham A [FA], Flitcham B [FB], Gorfield [G], Walsoken [W], Elm [E]) and seven cultivars (Cox [C], Fortune [F], Worcester [W], Grenadier [G], Bramley [B], Howgate Wonder [HW], Lord Derby [LD]) in East Anglia.

| Species | Bryophyte group | Epiphyte type | Orchard | Cultivar |
| --- | --- | --- | --- | --- |
| *Amblystegium serpens* (Hedw.) Schimp.^#^ | Moss | Facultative | FA, FB, G, W, E | C, F, W, G, B, LD |
| *Brachythecium rutabulum* (Hedw.) Schimp.^#^ | Moss | Facultative | FA, FB, G, W, E | C, F, W, G, B, HW, LD |
| *Brachytheciastrum velutinum* (Hedw.) Ignatov & Huttunen | Moss | Facultative | FA, G, E | C, B, LD |
| *Bryoerythrophyllum recurvirostrum* (Hedw.) P.C.Chen | Moss | Facultative | FA | F |
| *Bryum argenteum* Hedw. | Moss | Facultative | FA, FB, E | C, F, W, LD, B |
| *Bryum capillare* Hedw.^#^ | Moss | Facultative | FA, FB, G, W, E | C, F, W, G, B, LD |
| *Bryum moravicum* Podp. | Moss | Obligate | FA, FB, E | F, C, W, B |
| *Campylopus introflexus* (Hedw.) Brid. | Moss | Facultative | FA, FB, G, E | C, F, W, G, B |
| *Ceratodon purpureus* (Hedw.) Brid.^#^ | Moss | Facultative | FA, FB, G, W, E | C, F, W, G, B, LD |
| *Cryphaea heteromalla* (Hedw.) D. Mohr | Moss | Obligate | FA, FB, G | C, F, G, B |
| *Dicranum scoparium* Hedw. | Moss | Facultative | FA | C |
| *Dicranoweisia cirrata* (Hedw.) Lindb.^#^ | Moss | Facultative | FA, FB, G, W, E | C, F, W, G, B, HW, LD |
| *Frullania dilatata* (L.) Dumort. | Liverwort | Obligate | FA, FB, G, E | C, F, W, G, B, LD |
| *Grimmia pulvinata* (Hedw.) Sm.^#^ | Moss | Facultative | FA, FB, G, W, E | C, F, W, G, B, HW, LD |
| *Homalothecium sericeum* (Hedw.) Schimp. | Moss | Facultative | FA, FB, G, W, E | C, F, W, G, B, LD |
| *Hypnum cupressiforme agg.* Hedw.^#^ | Moss | Facultative | FA, FB, G, W, E | C, F, W, G, B, HW, LD |
| *Isothecium alopecuroides* (Dubois) Isoviita | Moss | Obligate | FA | C |
| *Isothecium myosuroides* Brid. | Moss | Obligate | FA, E | F, B |
| *Kindbergia praelonga* (Hedw.) Ochyra^#^ | Moss | Facultative | FA, FB, G, W, E | C, F, W, G, B, HW, LD |
| *Leptodictyum riparium* (Hedw.) Warnst. | Moss | Facultative | FA, G, E | C, F, G, B, LD |
| *Leskea polycarpa* Hedw. | Moss | Obligate | FA, G, E | C, F, G, B, LD |
| *Leucodon sciuroides* (Hedw.) Schwägr. | Moss | Obligate | FA | F |
| *Metzgeria furcata* (L.) Dumort.^#^ | Liverwort | Obligate | FA, G | F, G |
| *Orthotrichum affine* Schrad. ex Brid.^#^ | Moss | Obligate | FA, FB, G, W, E | C, F, W, G, B, LD |
| *Orthotrichum diaphanum* Brid.^#^ | Moss | Facultative | FA, FB, G, W, E | C, F, W, G, B, LD |
| *Orthotrichum lyellii* Hook. & Taylor | Moss | Obligate | FA, FB, G, W, E | C, F, G, B, LD |
| *Orthotrichum stramineum* Hornsch. ex Brid. | Moss | Obligate | FA, FB | C, F, W |
| *Pylaisia polyantha* (Hedw.) Schimp. | Moss | Obligate | E | LD |
| *Radula complanata* (L.) Dumort. | Liverwort | Obligate | FA, FB, E | F, C, W, LD, B |
| *Rhynchostegium confertum* (Dicks.) Schimp.^#^ | Moss | Facultative | FA, FB, G, W, E | C, F, W, G, B, HW, LD |
| *Sanionia uncinata* (Hedw.) Loeske | Moss | Obligate | FA | F |
| *Syntrichia laevipila* Brid. | Moss | Obligate | FA, FB, E | C, F, W, LD |
| *Syntrichia montana* Nees | Moss | Facultative | FA, FB, G, W, E | C, F, W, G, B, LD |
| *Syntrichia papillosa* Wilson Jur. | Moss | Obligate | FA, FB, E | C, F, W, LD, B |
| *Syntrichia ruralis* (Hedw.) F.Weber & D.Mohr | Moss | Facultative | FA, FB | C |
| *Syntrichia virescens* (De Not.) Ochyra | Moss | Facultative | FA, FB, G, E | C, F, W, G, LD, B |
| *Tortula muralis* Hedw. | Moss | Facultative | FA, FB, G, E | C, F, W, G, LD |
| *Ulota bruchii agg.* Hornsch. ex Brid. | Moss | Obligate | FA, FB, G, W, E | C, F, W, G, B, LD |
| *Ulota phyllantha* Brid. | Moss | Obligate | FA, FB | C, F, W |
| *Zygodon conoideus* (Dicks.) Hook. & Taylor | Moss | Obligate | FA, FB, E | C, F, W, LD, B |
| *Zygodon viridissimus** (Dicks.) Brid.^#^ | Moss | Facultative | FA, FB, E | C, F, W, B |

*may include records for *Z. rupestre*.

^#^ Species where cultivar is an important factor explaining distribution.

Appendix 2. Summary table of the linear model of species richness, where orchard and cultivar nested within orchard were factors.

|  | Df | Sum of Squares | Mean of Squares | F value | P value |
| --- | --- | --- | --- | --- | --- |
| Orchard | 4 | 5663.6 | 1415.89 | 358.174 | < 2.2e-16 |
| Orchard/cultivar | 5 | 413.4 | 82.68 | 20.915 | < 2.2e-16 |
| Residuals | 607 | 2399.5 | 3.95 |  |  |

Appendix 3. Summary plot of the permutation tests (10,000 randomisations) undertaken on the distance matrix of epiphyte bryophyte communities on each tree sampled where orchard and cultivar were factors tested sequentially.

|  | Df | Sum of Squares | Mean Sum of Squares | F model | R^2^ | P value |
| --- | --- | --- | --- | --- | --- | --- |
| Orchard | 4 | 19.32 | 4.83 | 26.36 | 0.16 | 0.0001 |
| Cultivar | 5 | 5.09 | 1.02 | 5.55 | 0.04 | 0.0001 |
| Residuals | 528 | 96.72 | 0.18 |  | 0.80 |  |
| Total | 537 | 121.12 |  |  | 1.0 |  |

Appendix 4. Stress plot for the NMDS on the epiphytic bryophyte community data. Stress = 0.2.

Appendix 5. R code used for analyses

**1) Analysis of species richness data**

#data file New_moss.csv (doi:10.5061/dryad.mb0sh)

spp_rich <- lm(SPP.RICH ~ Orchard + Orchard:Variety, moss_spp)

plot(spp_rich)

summary(spp_rich)

anova(spp_rich)

car::Anova(spp_rich)

summary(moss_spp$Orchard)

summary(moss_spp$Variety)

moss_spp$Orchard<-factor(moss_spp$Orchard)

moss_spp$Variety<-factor(moss_spp$Variety)

env <- moss_spp[, 2:3]

spp_rich_data <- cbind(env, moss_spp$SPP.RICH)

library(plyr)

spp_data <- rename(spp_rich_data, c("moss_spp$SPP.RICH" = "spp"))

summarySE <- function(data=NULL, measurevar, groupvars=NULL, na.rm=FALSE,

conf.interval=.95, .drop=TRUE) {

require(plyr)

# New version of length which can handle NA's: if na.rm==T, don't count them

length2 <- function (x, na.rm=FALSE) {

if (na.rm) sum(!is.na(x))

else length(x)

}

# This does the summary. For each group's data frame, return a vector with

# N, mean, and sd

datac <- ddply(data, groupvars, .drop=.drop,

.fun = function(xx, col) {

c(N = length2(xx[[col]], na.rm=na.rm),

mean = mean (xx[[col]], na.rm=na.rm),

sd = sd (xx[[col]], na.rm=na.rm)

)

},

measurevar

)

# Rename the "mean" column

datac <- rename(datac, c("mean" = measurevar))

datac$se <- datac$sd / sqrt(datac$N) # Calculate standard error of the mean

# Confidence interval multiplier for standard error

# Calculate t-statistic for confidence interval:

# e.g., if conf.interval is .95, use .975 (above/below), and use df=N-1

ciMult <- qt(conf.interval/2 + .5, datac$N-1)

datac$ci <- datac$se * ciMult

return(datac)

}

SR <- summarySE(spp_data, measurevar="spp", groupvars=c("Orchard","Variety"))

SR

library(ggplot2)

orchard_fig1 <- ggplot(SR, aes(x=Orchard, y=spp, fill=Variety)) +

geom_bar(position="dodge", stat="identity",

colour="black", # Use black outlines,

size=.3) + # Thinner lines

geom_errorbar(aes(ymin=spp-ci, ymax=spp+ci),

width=.2, # Width of the error bars

position=position_dodge(.9)) +

xlab("Cultivar in Orchard") +

ylab("Mean species richness per tree") +

scale_fill_manual(values=c("#E69F00", "#56B4E9", "#009E73", "#999999", "#0072B2", "#D55E00", "#CC79A7"), labels=c("Bramley", "Cox", "Fortune", "Grenadiers", "Howgate Wonder", "Lord Derby", "Worcester"))+

labs(fill="Cultivar")+

ggtitle("") +

scale_y_continuous(breaks=seq(0,12, by=2),expand = c(0,0), limits=c(0,12)) +

scale_x_discrete(breaks=c("Elm", "FlitchamA", "FlitchamB", "Gorfield", "Walsoken"), labels=c("Elm", "Flitcham A", "Flitcham B", "Gorfield", "Walsoken"))+

theme_bw()+

theme(panel.background = element_blank(),

panel.grid.major = element_blank(), #remove major-grid labels

panel.grid.minor = element_blank(), #remove minor-grid labels

plot.background = element_blank(), axis.text.x=element_text(angle=30, hjust=1, vjust=1))

orchard_fig1

**2) NMDS and permutation tests**

write.csv (moss,"moss_zero.csv", na="0")#data file New_moss.csv (doi:10.5061/dryad.mb0sh)

moss <- read.csv("moss_zero.csv", header=T, row.names=1)

#Define 'Orchard' and 'Variety' as factors

moss$Orchard<-factor(moss$Orchard)

moss$Variety<-factor(moss$Variety)

#Create two dataframes as objects for ordination, specifying [rownumbers, columnnumbers]

env<-moss[1:617, 2:3] #environmental variables ('Orchard' and 'Variety')

spp<-moss[1:617, 6:46] #species matrix

moss_join <- cbind(env,spp)

mossclean <- unique(moss_join)

write.csv(mossclean, "mossclean.csv")

mossclean <- read.csv("mossclean.csv", header=T, row.names=1)

mossclean2 <- mossclean[-c(399, 426), ]

write.csv(mossclean2, "mossclean2.csv")

envclean <- mossclean2[1:538, 1:2]

sppclean <- mossclean2[1:538, 3:43]

summary(envclean$Orchard:envclean$Variety)

library(vegan)

trans_orch <- wisconsin(sqrt(sppclean))

trans_nmds <-metaMDS(trans_orch, distance = "jaccard", autotransform = FALSE)

trans_nmds

stressplot(trans_nmds)

orch_ad <- adonis(trans_orch ~ Orchard + Variety, envclean, permutations = 10000, method = "jaccard")

orch_ad

#Add location as an additional factor

Location <- function(x) {

if(x=="FlitchamA"|x=="FlitchamB") y <- "F"

if(x=="Elm"|x=="Gorfield"|x=="Walsoken") y<-"W"

return(y)

}

envclean$Location <- sapply(envclean$Orchard, Location)

orch_adrev <- adonis(trans_orch ~ Location + Orchard + Variety, envclean, permutations = 10000, method = "jaccard")

orch_adrev

#Following instructions at http://chrischizinski.github.io/rstats/2014/04/13/vegan-ggplot2/

data.scores <- as.data.frame(scores(trans_nmds)) #Using the scores function from vegan to extract the site scores and convert to a data.frame

data.scores$site <- rownames(data.scores) # create a column of site names, from the rownames of data.scores

nmds_plot_data <- cbind(envclean,data.scores) #join environmental data to nmds scores

head(nmds_plot_data) #look at the data

species.scores <- as.data.frame(scores(trans_nmds, "species")) #Using the scores function from vegan to extract the species scores and convert to a data.frame

species.scores$species <- rownames(species.scores) # create a column of species, from the rownames of species.scores

head(species.scores) #look at the data

Both_plot <- ggplot() +

geom_point(data=nmds_plot_data,aes(x=NMDS1,y=NMDS2,shape=Orchard,colour=Variety),size=3) + # add the point markers

scale_shape_manual(values=c(0, 2, 4, 6, 8), labels=c("Elm", "Flitcham A", "Flitcham B", "Gorefield", "Walsoken")) +

scale_colour_manual(values=c("#E69F00", "#56B4E9", "#009E73", "#999999", "#0072B2", "#D55E00", "#CC79A7"), labels=c("Bramley", "Cox", "Fortune", "Grenadiers", "Howgate Wonder", "Lord Derby", "Worcester"))+

labs(colour="Cultivar")+

theme_bw()+

theme(axis.text.x = element_blank(), # remove x-axis text

axis.text.y = element_blank(), # remove y-axis text

axis.ticks = element_blank(), # remove axis ticks

axis.title.x = element_text(size=18), # remove x-axis labels

axis.title.y = element_text(size=18), # remove y-axis labels

panel.background = element_blank(),

panel.grid.major = element_blank(), #remove major-grid labels

panel.grid.minor = element_blank(), #remove minor-grid labels

plot.background = element_blank(), legend.text=element_text(size=10))

Both_plot

**3) Species level mixed model analysis**

moss <- read.csv("moss_zero.csv", header=T, row.names=1) #data file created above

library(lme4)

library(car)

#1

Am_sernull<-glmer(Am_ser~1+(1|Orchard), family=binomial(link="logit"), data=moss)

Am_serfull<-glmer(Am_ser~1+(1|Orchard/Variety), family=binomial(link="logit"), data=moss)

anova(Am_sernull, Am_serfull)

summary(Am_serfull)

#p=7.94e-05

#2

Br_rutnull<-glmer(Br_rut~1+(1|Orchard), family=binomial(link="logit"), data=moss)

Br_rutfull<-glmer(Br_rut~1+(1|Orchard/Variety), family=binomial(link="logit"), data=moss)

anova(Br_rutnull, Br_rutfull)

summary(Br_rutfull)

#p=7.39e-08 raw

#3

Br_velnull<-glmer(Br_vel~1+(1|Orchard), family=binomial(link="logit"), data=moss)

Br_velfull<-glmer(Br_vel~1+(1|Orchard/Variety), family=binomial(link="logit"), data=moss)

anova(Br_velnull, Br_velfull)

#0.03884

#4

Br_erynull<-glmer(Br_ery~1+(1|Orchard), family=binomial(link="logit"), data=moss)

Br_eryfull<-glmer(Br_ery~1+(1|Orchard/Variety), family=binomial(link="logit"), data=moss)

anova(Br_erynull, Br_eryfull)

#1

#5

Br_argnull<-glmer(Br_arg~1+(1|Orchard), family=binomial(link="logit"), data=moss)

Br_argfull<-glmer(Br_arg~1+(1|Orchard/Variety), family=binomial(link="logit"), data=moss)

anova(Br_argnull, Br_argfull)

#1

#6

Br_capnull<-glmer(Br_cap~1+(1|Orchard), family=binomial(link="logit"), data=moss)

Br_capfull<-glmer(Br_cap~1+(1|Orchard/Variety), family=binomial(link="logit"), data=moss)

anova(Br_capnull, Br_capfull)

summary(Br_capfull)

#0.0003608

#7

Br_mornull<-glmer(Br_mor~1+(1|Orchard), family=binomial(link="logit"), data=moss)

Br_morfull<-glmer(Br_mor~1+(1|Orchard/Variety), family=binomial(link="logit"), data=moss)

anova(Br_mornull, Br_morfull)

#0.3485

#9

Ca_intnull<-glmer(Ca_int~1+(1|Orchard), family=binomial(link="logit"), data=moss)

Ca_intfull<-glmer(Ca_int~1+(1|Orchard/Variety), family=binomial(link="logit"), data=moss)

anova(Ca_intnull, Ca_intfull)

#0.002051

#10

Ce_purnull<-glmer(Ce_pur~1+(1|Orchard), family=binomial(link="logit"), data=moss)

Ce_purfull<-glmer(Ce_pur~1+(1|Orchard/Variety), family=binomial(link="logit"), data=moss)

anova(Ce_purnull, Ce_purfull)

summary(Ce_purfull)

#0.000611

#11

Cr_hetnull<-glmer(Cr_het~1+(1|Orchard), family=binomial(link="logit"), data=moss)

Cr_hetfull<-glmer(Cr_het~1+(1|Orchard/Variety), family=binomial(link="logit"), data=moss)

anova(Cr_hetnull, Cr_hetfull)

#0.003671

#12

Di_cirnull<-glmer(Di_cir~1+(1|Orchard), family=binomial(link="logit"), data=moss)

Di_cirfull<-glmer(Di_cir~1+(1|Orchard/Variety), family=binomial(link="logit"), data=moss)

anova(Di_cirnull, Di_cirfull)

summary(Di_cirfull)

#1.067e-05

#13

Di_sconull<-glmer(Di_sco~1+(1|Orchard), family=binomial(link="logit"), data=moss)

Di_scofull<-glmer(Di_sco~1+(1|Orchard/Variety), family=binomial(link="logit"), data=moss)

anova(Di_sconull, Di_scofull)

summary(Di_scofull)

#1

#14

Fr_dilnull<-glmer(Fr_dil~1+(1|Orchard), family=binomial(link="logit"), data=moss)

Fr_dilfull<-glmer(Fr_dil~1+(1|Orchard/Variety), family=binomial(link="logit"), data=moss)

anova(Fr_dilnull, Fr_dilfull)

#0.1198

#15

Gr_pulnull<-glmer(Gr_pul~1+(1|Orchard), family=binomial(link="logit"), data=moss)

Gr_pulfull<-glmer(Gr_pul~1+(1|Orchard/Variety), family=binomial(link="logit"), data=moss)

anova(Gr_pulnull, Gr_pulfull)

summary(Gr_pulfull)

#7.943e-08

#16

Ho_sernull<-glmer(Ho_ser~1+(1|Orchard), family=binomial(link="logit"), data=moss)

Ho_serfull<-glmer(Ho_ser~1+(1|Orchard/Variety), family=binomial(link="logit"), data=moss)

anova(Ho_sernull, Ho_serfull)

#0.9857

#17

Hy_cup_agnull<-glmer(Hy_cup_ag~1+(1|Orchard), family=binomial(link="logit"), data=moss)

Hy_cup_agfull<-glmer(Hy_cup_ag~1+(1|Orchard/Variety), family=binomial(link="logit"), data=moss)

anova(Hy_cup_agnull, Hy_cup_agfull)

summary(Hy_cup_agfull)

#0.0006438

#18

Is_myonull<-glmer(Is_myo~1+(1|Orchard), family=binomial(link="logit"), data=moss)

Is_myofull<-glmer(Is_myo~1+(1|Orchard/Variety), family=binomial(link="logit"), data=moss)

anova(Is_myonull, Is_myofull)

#0.642

#19

Is_alonull<-glmer(Is_alo~1+(1|Orchard), family=binomial(link="logit"), data=moss)

Is_alofull<-glmer(Is_alo~1+(1|Orchard/Variety), family=binomial(link="logit"), data=moss)

anova(Is_alonull, Is_alofull)

#1

#20

Ki_pranull<-glmer(Ki_pra~1+(1|Orchard), family=binomial(link="logit"), data=moss)

Ki_prafull<-glmer(Ki_pra~1+(1|Orchard/Variety), family=binomial(link="logit"), data=moss)

anova(Ki_pranull, Ki_prafull)

summary(Ki_prafull)

#2.2e-16

#21

Le_ripnull<-glmer(Le_rip~1+(1|Orchard), family=binomial(link="logit"), data=moss)

Le_ripfull<-glmer(Le_rip~1+(1|Orchard/Variety), family=binomial(link="logit"), data=moss)

anova(Le_ripnull, Le_ripfull)

#1

#22

Le_polnull<-glmer(Le_pol~1+(1|Orchard), family=binomial(link="logit"), data=moss)

Le_polfull<-glmer(Le_pol~1+(1|Orchard/Variety), family=binomial(link="logit"), data=moss)

anova(Le_polnull, Le_polfull)

#1

#23

Le_scinull<-glmer(Le_sci~1+(1|Orchard), family=binomial(link="logit"), data=moss)

Le_scifull<-glmer(Le_sci~1+(1|Orchard/Variety), family=binomial(link="logit"), data=moss)

anova(Le_scinull, Le_scifull)

#0.2325

#24

Me_furnull<-glmer(Me_fur~1+(1|Orchard), family=binomial(link="logit"), data=moss)

Me_furfull<-glmer(Me_fur~1+(1|Orchard/Variety), family=binomial(link="logit"), data=moss)

anova(Me_furnull, Me_furfull)

summary(Me_furfull)

#0.0006035

#25

Or_affnull<-glmer(Or_aff~1+(1|Orchard), family=binomial(link="logit"), data=moss)

Or_afffull<-glmer(Or_aff~1+(1|Orchard/Variety), family=binomial(link="logit"), data=moss)

anova(Or_affnull, Or_afffull)

summary(Or_afffull)

#1.287e-07

#26

Or_dianull<-glmer(Or_dia~1+(1|Orchard), family=binomial(link="logit"), data=moss)

Or_diafull<-glmer(Or_dia~1+(1|Orchard/Variety), family=binomial(link="logit"), data=moss)

anova(Or_dianull, Or_diafull)

summary(Or_diafull)

#1.382e-12

#27

Or_lyenull<-glmer(Or_lye~1+(1|Orchard), family=binomial(link="logit"), data=moss)

Or_lyefull<-glmer(Or_lye~1+(1|Orchard/Variety), family=binomial(link="logit"), data=moss)

anova(Or_lyenull, Or_lyefull)

#1

#28

Or_strnull<-glmer(Or_str~1+(1|Orchard), family=binomial(link="logit"), data=moss)

Or_strfull<-glmer(Or_str~1+(1|Orchard/Variety), family=binomial(link="logit"), data=moss)

anova(Or_strnull, Or_strfull)

#0.9997

#29

Py_polnull<-glmer(Py_pol~1+(1|Orchard), family=binomial(link="logit"), data=moss)

Py_polfull<-glmer(Py_pol~1+(1|Orchard/Variety), family=binomial(link="logit"), data=moss)

anova(Py_polnull, Py_polfull)

#1

#30

Ra_comnull<-glmer(Ra_com~1+(1|Orchard), family=binomial(link="logit"), data=moss)

Ra_comfull<-glmer(Ra_com~1+(1|Orchard/Variety), family=binomial(link="logit"), data=moss)

anova(Ra_comnull, Ra_comfull)

#0.9155

#31

Rh_connull<-glmer(Rh_con~1+(1|Orchard), family=binomial(link="logit"), data=moss)

Rh_confull<-glmer(Rh_con~1+(1|Orchard/Variety), family=binomial(link="logit"), data=moss)

anova(Rh_connull, Rh_confull)

summary(Rh_confull)

#0.0003567

#32

Sa_uncnull<-glmer(Sa_unc~1+(1|Orchard), family=binomial(link="logit"), data=moss)

Sa_uncfull<-glmer(Sa_unc~1+(1|Orchard/Variety), family=binomial(link="logit"), data=moss)

anova(Sa_uncnull, Sa_uncfull)

#0.2325

#33

Sy_monnull<-glmer(Sy_mon~1+(1|Orchard), family=binomial(link="logit"), data=moss)

Sy_monfull<-glmer(Sy_mon~1+(1|Orchard/Variety), family=binomial(link="logit"), data=moss)

anova(Sy_monnull, Sy_monfull)

#0.1529

#34

Sy_laenull<-glmer(Sy_lae~1+(1|Orchard), family=binomial(link="logit"), data=moss)

Sy_laefull<-glmer(Sy_lae~1+(1|Orchard/Variety), family=binomial(link="logit"), data=moss)

anova(Sy_laenull, Sy_laefull)

#0.9993

#35

Sy_papnull<-glmer(Sy_pap~1+(1|Orchard), family=binomial(link="logit"), data=moss)

Sy_papfull<-glmer(Sy_pap~1+(1|Orchard/Variety), family=binomial(link="logit"), data=moss)

anova(Sy_papnull, Sy_papfull)

#0.003122

#36

Sy_rurnull<-glmer(Sy_rur~1+(1|Orchard), family=binomial(link="logit"), data=moss)

Sy_rurfull<-glmer(Sy_rur~1+(1|Orchard/Variety), family=binomial(link="logit"), data=moss)

anova(Sy_rurnull, Sy_rurfull)

#0.01382

#37

Sy_virnull<-glmer(Sy_vir~1+(1|Orchard), family=binomial(link="logit"), data=moss)

Sy_virfull<-glmer(Sy_vir~1+(1|Orchard/Variety), family=binomial(link="logit"), data=moss)

anova(Sy_virnull, Sy_virfull)

#0.1277

#38

To_murnull<-glmer(To_mur~1+(1|Orchard), family=binomial(link="logit"), data=moss)

To_murfull<-glmer(To_mur~1+(1|Orchard/Variety), family=binomial(link="logit"), data=moss)

anova(To_murnull, To_murfull)

#0.9969

#39

Ul_brunull<-glmer(Ul_bru~1+(1|Orchard), family=binomial(link="logit"), data=moss)

Ul_brufull<-glmer(Ul_bru~1+(1|Orchard/Variety), family=binomial(link="logit"), data=moss)

anova(Ul_brunull, Ul_brufull)

#0.157

#40

Ul_phynull<-glmer(Ul_phy~1+(1|Orchard), family=binomial(link="logit"), data=moss)

Ul_phyfull<-glmer(Ul_phy~1+(1|Orchard/Variety), family=binomial(link="logit"), data=moss)

anova(Ul_phynull, Ul_phyfull)

#0.9989

#41

Zy_connull<-glmer(Zy_con~1+(1|Orchard), family=binomial(link="logit"), data=moss)

Zy_confull<-glmer(Zy_con~1+(1|Orchard/Variety), family=binomial(link="logit"), data=moss)

anova(Zy_connull, Zy_confull)

#0.00369

#42

Zy_virnull<-glmer(Zy_vir~1+(1|Orchard), family=binomial(link="logit"), data=moss)

Zy_virfull<-glmer(Zy_vir~1+(1|Orchard/Variety), family=binomial(link="logit"), data=moss)

anova(Zy_virnull, Zy_virfull)

summary(Zy_virfull)

#9.412e-08

adjp2 <- p.adjust(c(7.94e-05, 7.39e-08, 0.03884, 1, 1, 0.0003608, 0.3485, 0.002051, 0.000611, 0.003671, 1.067e-05, 1, 0.1198, 7.943e-08, 0.9857, 0.0006438, 0.642, 1, 2.2e-16, 1, 1, 0.2325, 0.0006035, 1.287e-07, 1.382e-12, 1, 0.9997, 1, 0.9155, 0.0003567, 0.2325, 0.1529, 0.9993, 0.003122, 0.01382, 0.1277, 0.9969, 0.157, 0.9989, 0.00369, 9.412e-08))

adjp2
